# Supplementary material for: Interpretable multiple instance learning for hematologic diagnosis from peripheral blood smears
Source: Commun Med (Lond). 2026 Apr 15;6:309. doi: 10.1038/s43856-026-01558-x (PMC13216603; doi:10.1038/s43856-026-01558-x)
Supplement: Supplementary file 2 — Supplemental Information [file 43856_2026_1558_MOESM2_ESM.pdf]

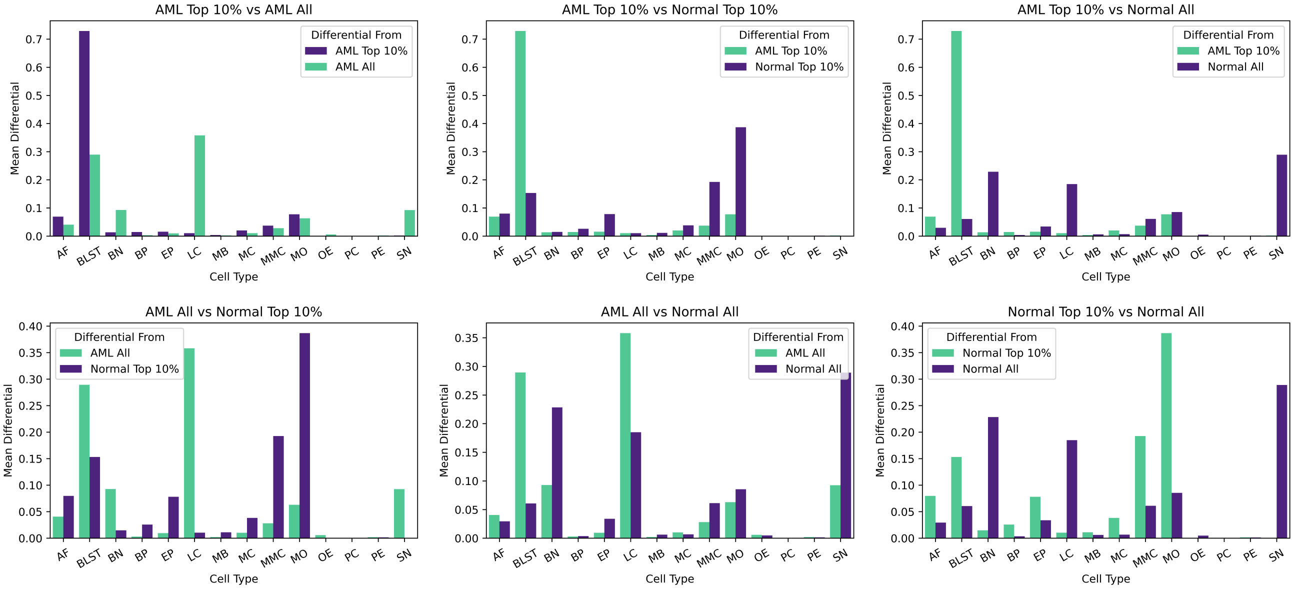

Figure Supplementary 1. **Comparative attention barplots** In each bar plot we are comparing the mean differentials of the patients taken from two differential groups. The groups are chosen by either selecting the cells from patients diagnosed with AML or Normal. Further for each disease category, the selected cells are ranked by their attention values from the CAREMIL model and either the top 10% of those cells or all the cells are used to create the mean differential. This comparison highlights the effectiveness of the CAREMIL model to assign attention values to the relevant cells in the diagnoses of the samples.

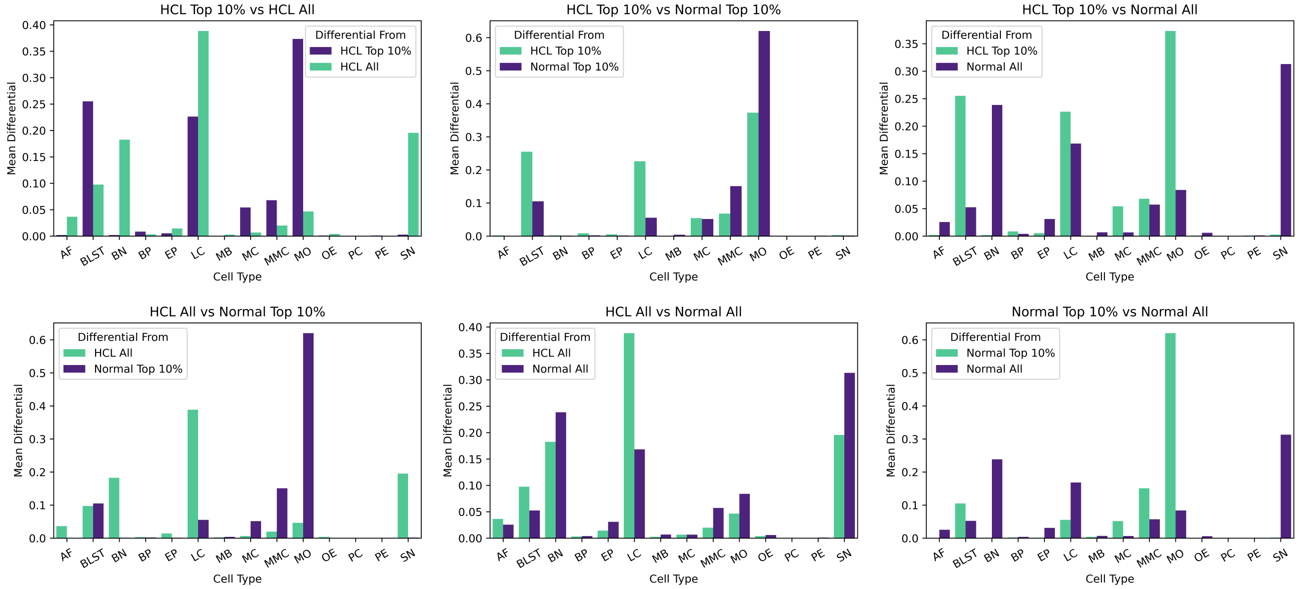

Figure Supplementary 2. **Comparative attention barplots** In each bar plot we are comparing the mean differentials of the patients taken from two differential groups. The groups are chosen by either selecting the cells from patients diagnosed with HCL or Normal. Further for each disease category, the selected cells are ranked by their attention values from the CAREMIL model and either the top 10% of those cells or all the cells are used to create the mean differential. This comparison highlights the effectiveness of the CAREMIL model to assign attention values to the relevant cells in the diagnoses of the samples.

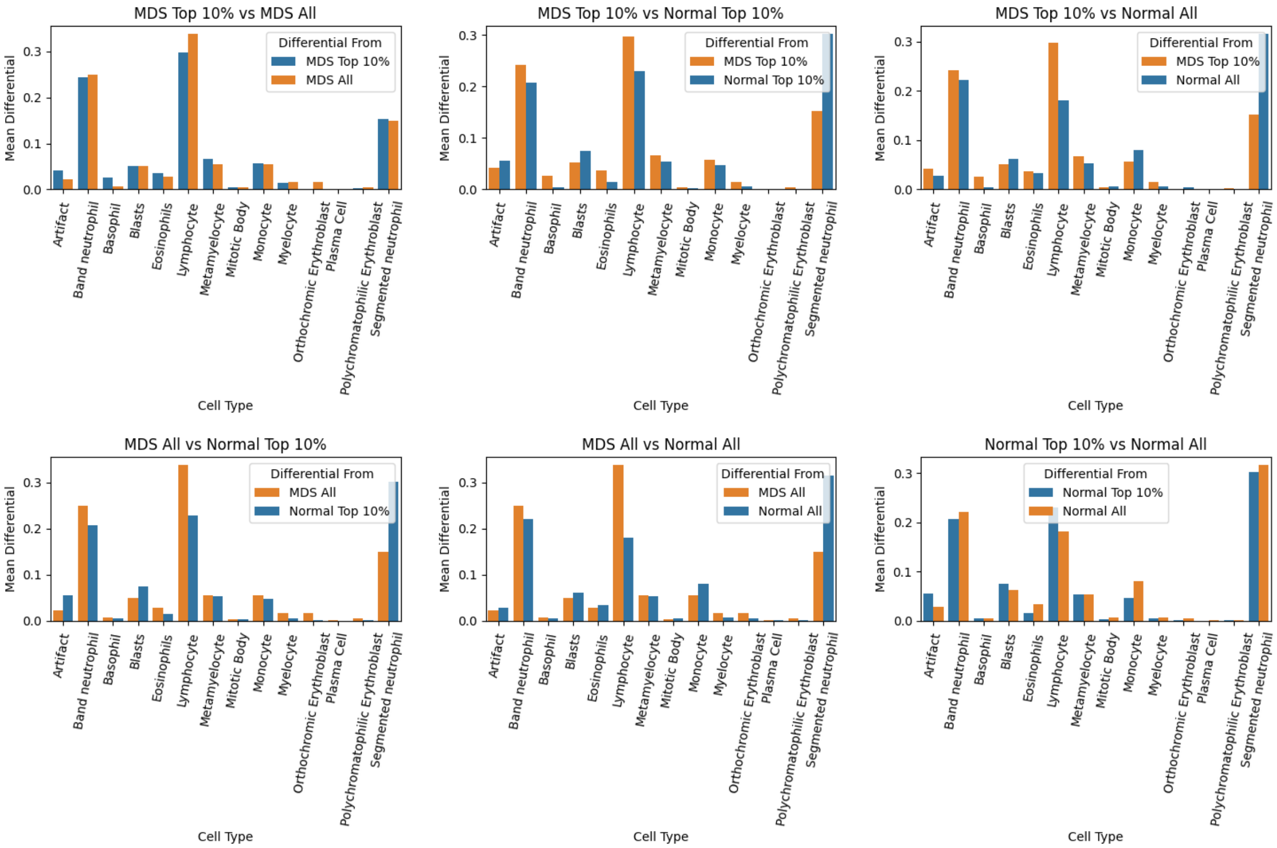

Figure Supplementary 3. Comparative attention barplots In each bar plot we are comparing the mean differentials of the patients taken from two differential groups. The groups are chosen by either selecting the cells from patients diagnosed with MDS or Normal. Further for each disease category, the selected cells are ranked by their attention values from the CAREMIL model and either the top 10% of those cells or all the cells are used to create the mean differential. This comparison highlights the effectiveness of the CAREMIL model to assign attention values to the relevant cells in the diagnoses of the samples.

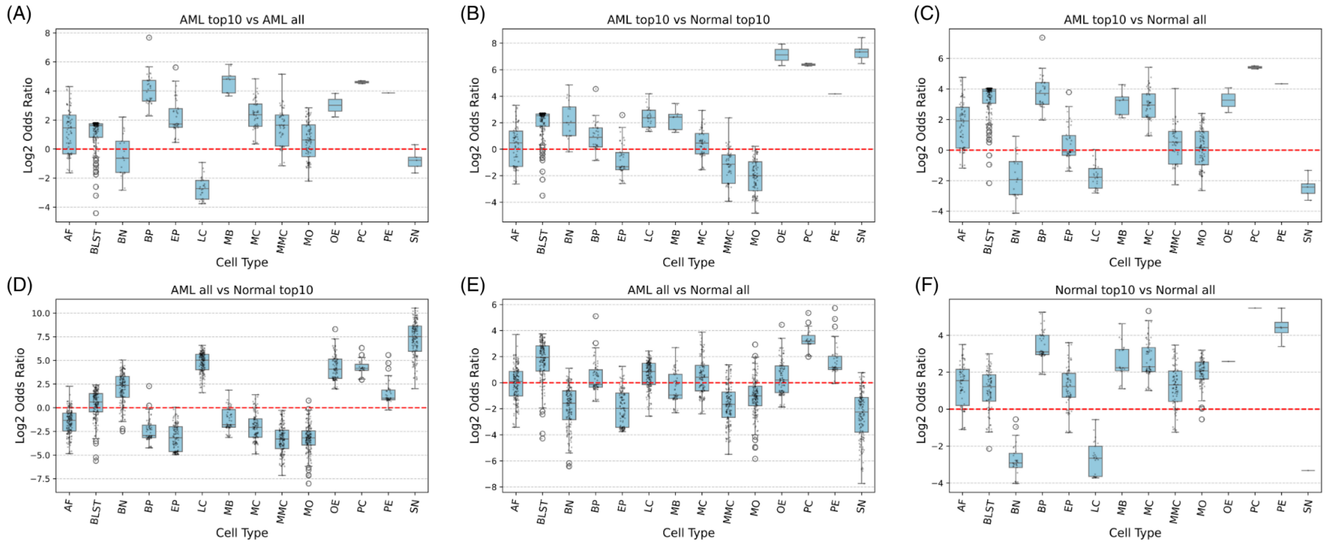

Figure Supplementary 4. Comparative Log odds ratios In each plot we are comparing the mean differentials of the patients taken from the the two differential types and plotting the log odds ratios of the two.

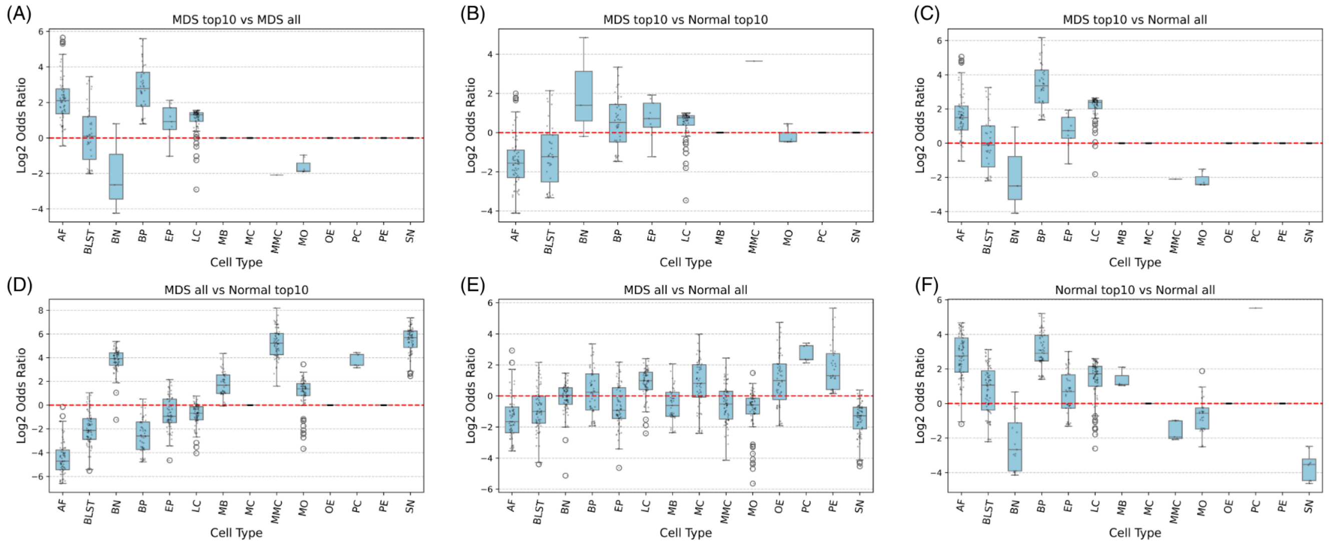

Figure Supplementary 5. Comparative Log odds ratios In each plot we are comparing the mean differentials of the patients taken just from the the two differential types and plotting the log odds ratios of the two.

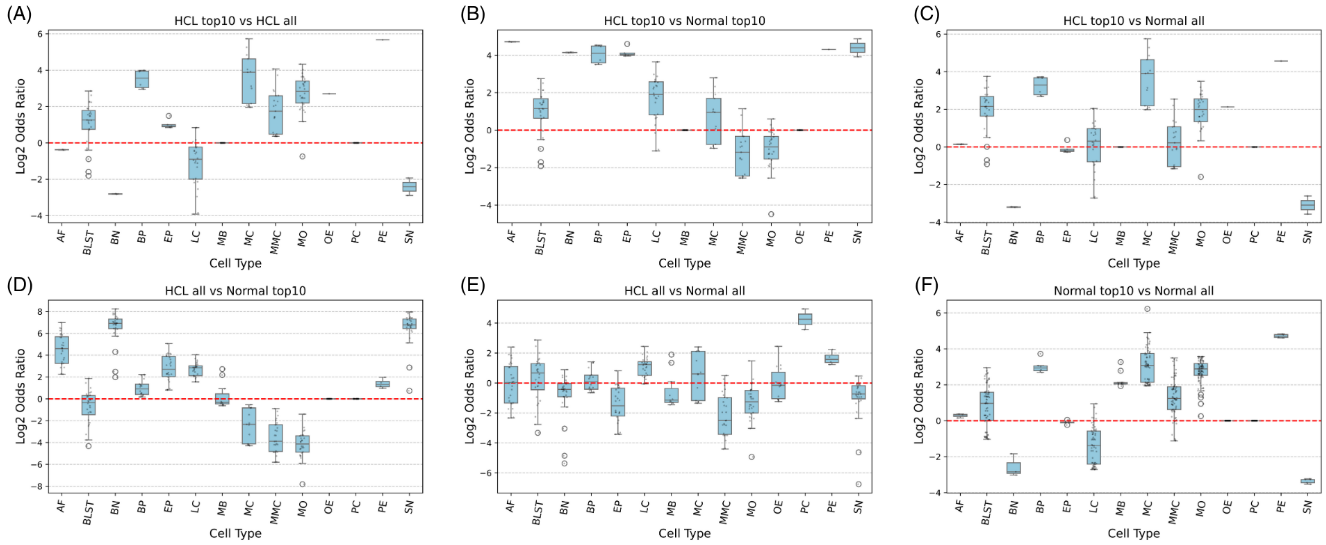

Figure Supplementary 6. Comparative Log odds ratios In each plot we are comparing the mean differentials of the patients taken just from the the two differential types and plotting the log odds ratios of the two.

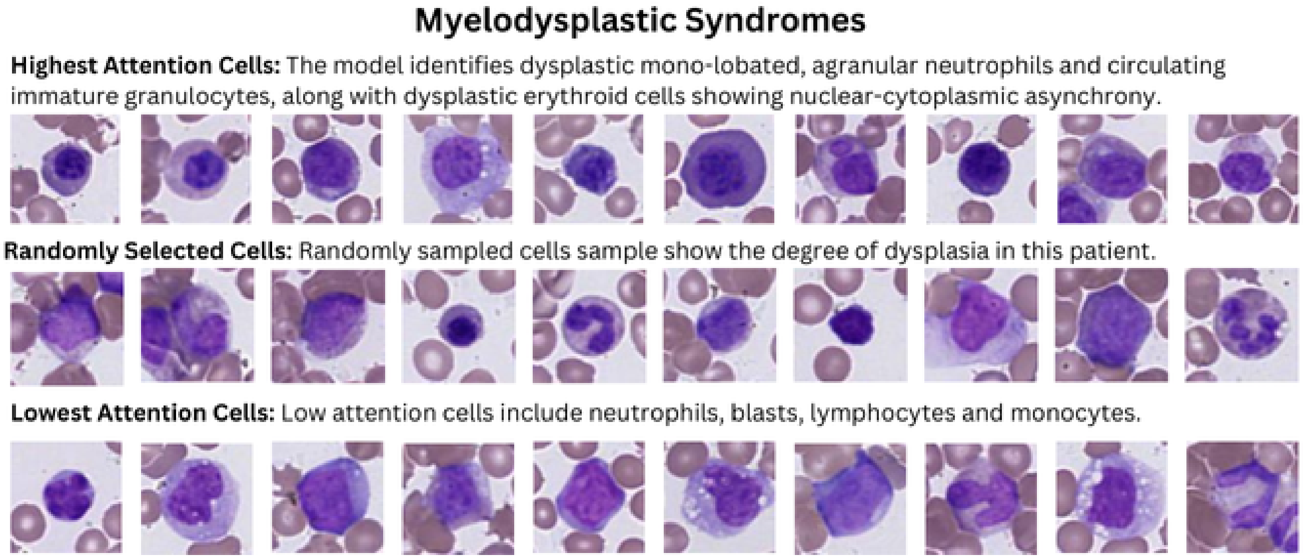

Figure Supplementary 7. **MDS Attention Panel** High-attention cells include dysplastic hypogranular neutrophils, monolobated neutrophils, dysplastic erythroblasts showing nuclear-cytoplasmic dyssynchrony, and abnormal immature granulocytes circulating in peripheral blood.

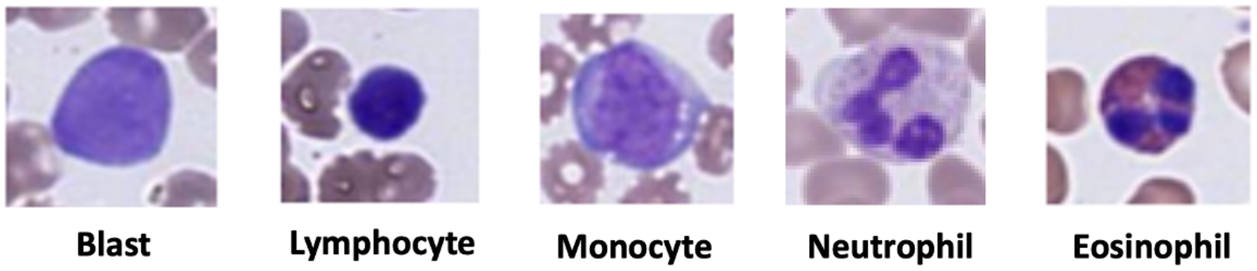

Figure Supplementary 8. Cell types seen in the blood. Five prototypical cell types seen in peripheral blood smears are shown. Blasts are stem-cell-like cells that are increased in number in acute myeloid and lymphoid leukemias. The purple nucleus makes up nearly the entire cell (high nuclear:cytoplasmic ratio). The nuclear chromatin is fine, and small circles (nucleoli) can be seen within it. The cell is relatively large with almost no visible blue cytoplasm. Lymphocytes have darker, more mature nuclear chromatin. Monocytes have visible folds in the nucleus and spongy chromatin, blue-gray cytoplasm, and sometimes visible white vacuoles in the cytoplasm. Neutrophils and eosinophils have segmented nuclei. The eosinophil is notable for its bright pink cytoplasm.

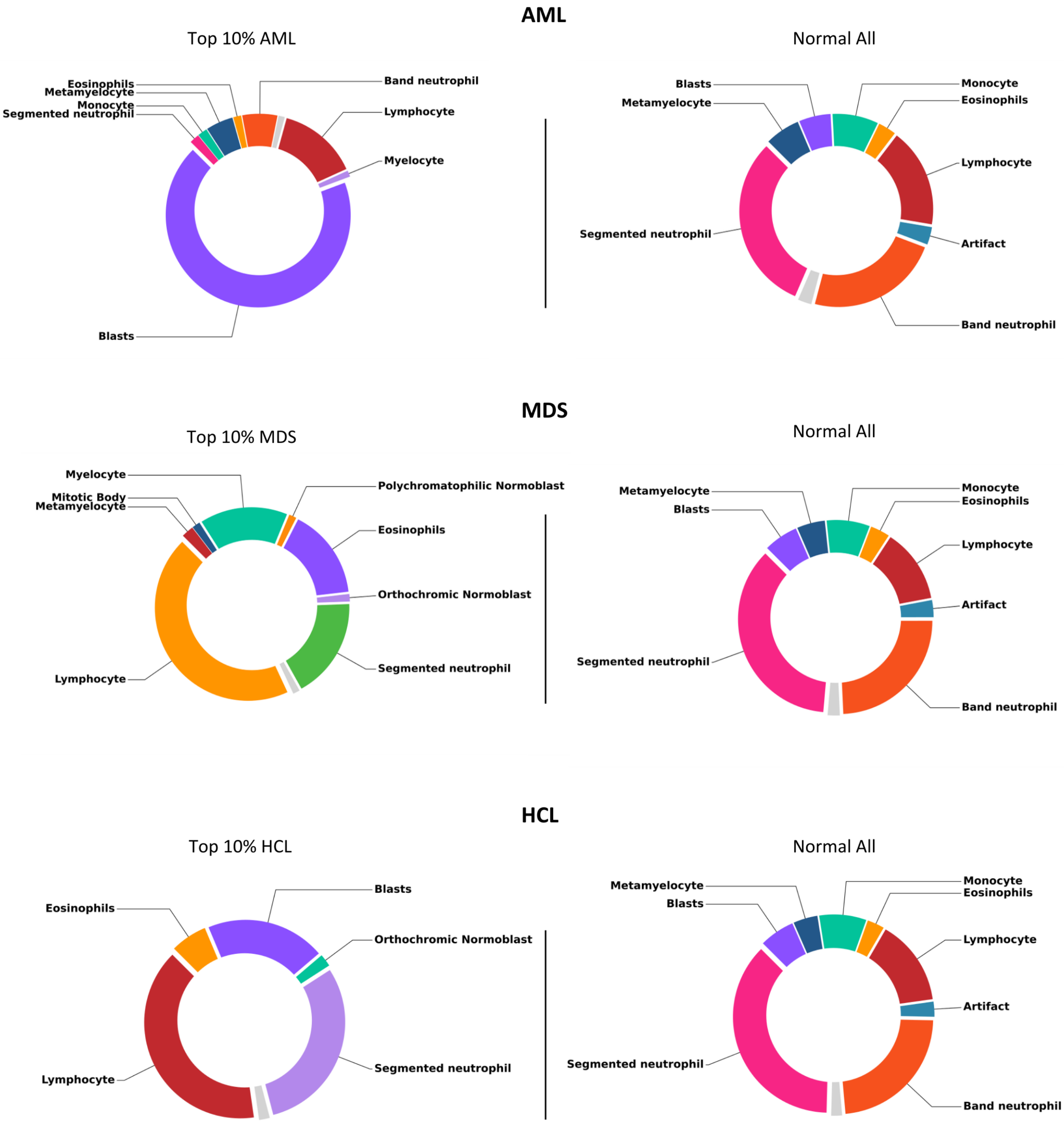

Figure Supplementary 9. **Comparative pie charts** In each plot we are comparing the mean differentials of the patients taken from the the disease patients' top 10% cells by attention value on the left and all the cells from the normal patient samples on the right.

Table Supplementary 1. Different cytometry-based ML classifiers and their hyperparameters

Classifiers	Hyperparameters	Values
SVC	kernel	rbf (radial basis function)
	error (c)	[5,10, 20, 50, 100, 200]
	Decision Functions	OvO, OvR
Random Forest	n_estimators	range(100,300,30)
	max_depth	[6,8,10,12]
	min_sample_split	[4,6,8,10]
Logistic Regression	Objective Function	[newton_cg, liblinear]
	C	[1,10,20,30]
Multinomial Naive Bayes	α	[0.1,1,10]
	Prior Probabilities	[True, False]
XG Boost	n_estimators	range(90,160,20)
	max_depth	range(3,8,1)
	learning rate	[0.02,0.05]
	γ	[0.05,0.1,0.2]
	Objective Function	softmax
Multi-Layer Perceptron	Activation Function	Relu
	Optimizer	Adam
	Hidden Layers	(32,64,32)

Table Supplementary 2. Hyperparameters for MIL models training

Hyperparameters	Values
Learning Rate	1e-4
Epochs	300
Learning Rate Scheduler	CyclicLR, mode=triangular2
Loss Function	Cross Entropy Loss
Batch Size	16
Optimizer	Adagrad
Train, Validation, Test Split	0.55, 0.2, 0.25
GPUs	3 (RTX 3090 each)
Trainable parameters in CAREMIL (This excludes the parameters in the encoder model)	1,419,202
Trainable parameters in Gated MIL	882,180
Trainable parameters in DSMIL	164,103
Parameters in ResNext50 (Deep Heme)	25 million
Parameters in ViT-Huge (UNI-2h and Virchow2)	632 million

Table Supplementary 3. Patient cohort by diagnosis

**Diagnosis**	**Number of Patients**
Acute Leukemia	307
Normal	130
Myelodysplastic syndromes	95
Hairy Cell Leukemia	41

Table Supplementary 4. Aggregation functions used in the analysis

Standard Deviation	*Mean*
$g(\{p_j\}) = \sqrt{\frac{1}{\lvert j\rvert}\sum_j (p_j - \bar{p})^2}$	$g(\{p_j\}) = \frac{1}{\lvert j\rvert}\sum_j p_j$
Generalized Mean	*Log Sum Exponentiation*
$g(\{p_j\}) = \left(\frac{1}{\lvert j\rvert}\sum_j p_j^r\right)^{\frac{1}{r}}$	$g(\{p_j\}) = \frac{1}{r}\log\left(\frac{1}{\lvert j\rvert}\sum_j e^{r\cdot p_j}\right)$

Table Supplementary 5. Summary of aggregation function experiments: Three groups of functions were experimented with for aggregation of cell embeddings. The performance difference across the different groups was negligible and attributed to stochasticity.

**Aggregation group**	**Aggregation function**
Normal	Mean, Standard Deviation, Max, Min
GM	Generalized mean with $r = 1.0, 2.5$ and 5.0
LSE	Log Sum Exponentiation with $r = 2.5$ and 5.0

Table Supplementary 6. Table of experiments: Summary of diagnostic experiments

Experiments	Description
AL vs NL	Binary classifier of acute leukemia vs normal
MDS vs NL	Binary classifier of myelodysplastic syndromes vs normal
HCL vs NL	Binary classifier of hairy cell leukemia
Multiclass	4-way classification between acute leukemia, myelodysplastic syndrome, hairy cell leukemia and normal

Table Supplementary 7. **Overview of Model Architectures and Performances. Model Architectures:** A summary description of each model architecture used in the study.

Model Name	Description
Cytometry-Based ML Classifiers	The DeepHeme classifier sorts the cells into respective classes to create a cytometric vector of the relative proportion of each cell class. This vector is used to train ML classifiers to predict the diagnosis.
Gated MIL - ImageNet	The cell embeddings are created from an ImageNet-trained model and passed to Gated MIL for predictions.
CAREMIL - ImageNet	The cell embeddings are created from an ImageNet-trained model and passed to CAREMIL for predictions.
DSMIL - ImageNet	The cell embeddings are created from an ImageNet-trained model and passed to DSMIL for predictions.
Gated MIL - Deep Heme	The cell embeddings from the DeepHeme model are passed to the Gated MIL model for predictions.
CAREMIL - Deep Heme	The cell embeddings from the DeepHeme model are passed to the CAREMIL model for predictions.
DSMIL - Deep Heme	The cell embeddings from the DeepHeme model and passed to DSMIL for predictions.
Gated MIL - UNI2-h	The cell embeddings from the UNI2-h model and passed to Gated MIL for predictions.
CAREMIL - UNI2-h	The cell embeddings from the UNI2-h model and passed to CAREMIL for predictions.
DSMIL - UNI2-h	The cell embeddings from the UNI2-h model and passed to DSMIL for predictions.
Gated MIL - Virchow2	The cell embeddings from the Virvchow2 model are passed to the Gated MIL model for predictions.
CAREMIL - Virchow2	The cell embeddings from the Virchow2 model are passed to the CAREMIL model for predictions.
DSMIL - Virchow2	The cell embeddings from the Virchow2 model and passed to DSMIL for predictions.

Table Supplementary 8. **Model Performance**

(A) AUROC Scores: AUROC scores with standard deviations for each model architecture across diagnostic experiments. The highest-performing models for each experiment are highlighted in bold.

Model Type - Encoder	AML vs NL	MDS vs NL	HCL vs NL	Multiclass
Cytometry-Based ML Classifiers	0.991 ± 0.00	0.877 ± 0.00	0.714 ± 0.00	0.892 ± 0.000
CAREMIL - Deep Heme	$\mathbf{0.999 \pm 0.002}$	0.891 ± 0.024	$\mathbf{0.962 \pm 0.009}$	0.916 ± 0.027
CAREMIL - Imagenet	0.750 ± 0.040	0.863 ± 0.066	0.766 ± 0.089	0.802 ± 0.046
CAREMIL - UNI2-h	0.884 ± 0.017	$\mathbf{0.896 \pm 0.009}$	0.764 ± 0.063	$\mathbf{0.923 \pm 0.023}$
CAREMIL - Virchow2	0.923 ± 0.016	0.758 ± 0.026	0.770 ± 0.083	0.873 ± 0.019
Gated MIL - Deep Heme	0.957 ± 0.010	0.829 ± 0.064	0.936 ± 0.061	0.919 ± 0.016
Gated MIL - Imagenet	0.770 ± 0.073	0.787 ± 0.066	0.700 ± 0.054	0.599 ± 0.062
Gated MIL - UNI2-h	0.773 ± 0.062	0.816 ± 0.088	0.767 ± 0.071	0.819 ± 0.033
Gated MIL - Virchow2	0.866 ± 0.005	0.823 ± 0.036	0.834 ± 0.015	0.806 ± 0.012
DSMIL - Deep Heme	0.972 ± 0.007	0.725 ± 0.089	0.803 ± 0.185	0.825 ± 0.026
DSMIL - Imagenet	0.416 ± 0.086	0.478 ± 0.064	0.404 ± 0.046	0.579 ± 0.029
DSMIL - UNI-2h	0.675 ± 0.069	0.700 ± 0.060	0.635 ± 0.091	0.670 ± 0.040
DSMIL - Virchow2	0.664 ± 0.075	0.680 ± 0.014	0.614 ± 0.071	0.664 ± 0.027

(B) F1 Scores: F1 scores with standard deviations for each model architecture across diagnostic experiments. The highest-performing models for each experiment are highlighted in bold. For multiclass we present the one-vs-rest f1 macro averaged score

Model Type - Encoder	AML vs NL	MDS vs NL	HCL vs NL	Multiclass
CAREMIL - Deep Heme	$\mathbf{0.942 \pm 0.017}$	$\mathbf{0.798 \pm 0.045}$	$\mathbf{0.839 \pm 0.048}$	0.679 ± 0.066
CAREMIL - Imagenet	0.406 ± 0.029	0.413 ± 0.050	0.478 ± 0.056	0.453 ± 0.098
CAREMIL - UNI2-h	0.820 ± 0.022	0.747 ± 0.007	0.704 ± 0.095	0.629 ± 0.039
CAREMIL - Virchow2	0.691 ± 0.128	0.756 ± 0.053	0.625 ± 0.132	0.600 ± 0.048
Gated MIL - Deep Heme	0.910 ± 0.024	0.743 ± 0.048	$\mathbf{0.839 \pm 0.084}$	$\mathbf{0.696 \pm 0.033}$
Gated MIL - Imagenet	0.624 ± 0.044	0.626 ± 0.008	0.571 ± 0.080	0.368 ± 0.055
Gated MIL - UNI2-h	0.642 ± 0.046	0.714 ± 0.051	0.572 ± 0.024	0.471 ± 0.058
Gated MIL - Virchow2	0.709 ± 0.055	0.481 ± 0.074	0.662 ± 0.079	0.513 ± 0.036
DSMIL - Deep Heme	0.869 ± 0.029	0.753 ± 0.095	0.668 ± 0.036	0.515 ± 0.062
DSMIL - Imagenet	0.442 ± 0.053	0.323 ± 0.022	0.546 ± 0.087	0.307 ± 0.023
DSMIL - UNI-2h	0.607 ± 0.081	0.573 ± 0.066	0.445 ± 0.072	0.404 ± 0.073
DSMIL - Virchow2	0.596 ± 0.038	0.590 ± 0.053	0.495 ± 0.035	0.380 ± 0.065
