# Supplementary material for: Interpretable multiple instance learning for hematologic diagnosis from peripheral blood smears
Source: Commun Med (Lond). 2026 Apr 15;6:309. doi: 10.1038/s43856-026-01558-x (PMC13216603; doi:10.1038/s43856-026-01558-x)
Supplement: Supplementary file 3 — Description of Additional Supplementary Files [file 43856_2026_1558_MOESM3_ESM.docx]

**Description of Additional Supplementary Files**

File name: Supplementary Data 1

Description: This csv contains the data to create Figure 3. Each row represents a synthetic patient created by merging blast cells from the AML patients with normal cells from the Normal patients. The columns of the csv are:

- Patient Name: Synthetic patient name for each row

- Doping Level: Percentage of blast cells mixed with normal cells

- Predicted label: Predicted label of the synthetic patient on a probability threshold of 0.5.

- AML: Predicted probability of AML from CAREMIL model

- Normal: Predicted probability of Normal from CAREMIL model

File name: Supplementary Data 2

Description: This is the csv to create figure 4. For each sub figure, 4a, 4b, 4c, we use <disease>_Top10% and <disease>_All for the 3 diseases aml, mds and hcl.

The 'Mean' value in the csv represents the percentage differential for that 'Cell Type'. The mean value is computed by calculating the percentage differential for every patient and then averaging it out across all patients.

The 'Differential From' column tells which group of patients was this average calculated over.
